# Highlighting the forgotten: Tuberculosis amidst the humanitarian crisis and COVID-19 in Afghanistan

**DOI:** 10.1016/j.amsu.2022.103671

**Published:** 2022-04-28

**Authors:** Mohammad Yasir Essar, Arash Nemat, Shoaib Ahmad, Ahsan Zil-E-Ali, Roy Rillera Marzo, Michael Head

**Affiliations:** Afghanistan National Charity Organization for Special Diseases (ANCOSD), Kabul, Afghanistan; Kabul University of Medical Sciences, Kabul, Afghanistan; Kabul University of Medical Sciences, Kabul, Afghanistan; District Head Quarter Teaching Hospital, Faisalabad, Pakistan; Pennsylvania State University, Hershey, PA, United States; Department of Community Medicine, International Medical School, Management and Science University, Shah Alam, Selangor, Darul Ehsan, Malaysia; Clinical Informatics Research Unit, Faculty of Medicine, University of Southampton, Southampton, United Kingdom

In Afghanistan, there is an ongoing humanitarian crisis that will have severe consequences on the health of the Afghan population. There are estimates that 1 million children may die of malnutrition across 2022. Factors such as the lack of basic sanitation, a fragile healthcare system and limited access to medicines will also contribute to a significant and likely excess burden of disease over the coming years.

Afghanistan is a country with a huge burden of respiratory health diseases, including tuberculosis [1]. According to the Global Tuberculosis report, Afghanistan is one of the countries with the highest incidence of tuberculosis in the WHO Eastern Mediterranean Region (EMRO). There are an estimated annual 13 thousand Afghans die due to tuberculosis. In 2016, over 65,000 cases and 11,000 deaths were registered because of TB [2]. In 2018, Afghanistan reported 48,420 TB cases out of the estimated 70,000 incident cases [3]. According to Afghanistan's National TB Program (NTP), in 2019 over 48,420 TB cases were diagnosed and reported [4]. In 2020, the first year of the COVID-19 pandemic, the Ministry of Public Health of Afghanistan reported 50,000 new tuberculosis cases with 10,000 deaths. However, WHO estimates indicate that this is likely to be significant under-reporting, with a probable 73,000 people had tuberculosis in 2020 in Afghanistan [5].

Worldwide, the COVID-19 pandemic has disrupted the surveillance and management of TB programs. Following a large increase of cases between 2017 and 2019, there was a fall of 18% between 2019 and 2020, from 7.1 million to 5.8 million. Additionally, the global number of people who were provided with TB preventive treatment increased from 1.0 million in 2015 to 3.6 million in 2019, but this positive trend was reversed in 2020, with a 21% reduction to 2.8 million [6]. The Global Fund estimates that was a 19% decrease in the number of people treated for drug-resistant tuberculosis in the countries receiving Global Fund support and a 37% decrease in people treated for extensively drug-resistant tuberculosis [7].

In Afghanistan, the country's healthcare system diverted significant healthcare resources to the containment of the pandemic. A consequence of that is that other infectious diseases like polio spiked, due to this diversion [8]. To date, Afghanistan has entered into the third wave of the pandemic. As of 16th of April 2022, 178, 387 cases and 7676 deaths are registered [9]. The true number of cases will undoubtedly be higher than the official reported count with under-reporting due to political unrest, and a lack of a comprehensive surveillance system.

Given that the limited availability of healthcare resources, the cases of tuberculosis in Afghanistan have significantly increased in comparison to the previous years. The history of the Taliban government gives great cause for concern around the intricate sociopolitical affairs of the country and how this will impact upon both healthcare, and healthcare access for specific demographics. For example, there are serious human rights concerns around access to healthcare for women. The burdens of tuberculosis globally are typically greater in males than females, but the opposite is true in Afghanistan [10].

Cultural barriers and the resulting stigma is another major problem that the national healthcare system and international organizations such as the WHO are facing in Afghanistan [10]. There is observed stigma whereby some tuberculosis patients consider the disease a disgrace, and tend to avoid seeking medical attention, delaying diagnosis and worsening the condition [11]. This is more common in women, and in rural areas where education, healthcare access and health promotion is limited. It is plausible that individuals may have concerns about ending up as a COVID patient if they will confess having any problem related to the respiratory system.

Another difficulty faced by Afghanistan is the high prevalence of Drug-Resistant tuberculosis, another consequence of delaying or stopping treatment [2]. Tuberculosis is treatable, but drug-resistant or multi drug-resistant forms are complex to manage and have higher mortality rates.

Currently, Afghanistan is in the midst of a humanitarian crisis after the U.S and international allies' withdrawal [12]. In the aftermath of this, including financial isolation from the global system and barriers to humanitarian organizations, the country faces terrible difficulties. The largest health services provider ‘Sehetmandi’ came into halt, functioning at only 17% of its previous capacity [13]. Over the years, the healthcare system relied on external funding from World Bank, and other international organizations. An immediate suspension of funding after the withdrawal, caused significant problems to the crumbling healthcare system. Healthcare workers have not been paid for months. There has been a dire increase in malnutrition among children. In addition to this, a huge economic crisis has blighted the population [14].

At the midst of this humanitarian crisis, efforts should be continued for the containment of major health diseases, including tuberculosis. Cooperations with the Taliban's government are difficult and sensitive; however, they should make efforts to coordinate with the NGOs within and outside of Afghanistan to ensure cumulative efforts of years of achievement are not lost. The National Tuberculosis (TB) Control Program (NTP) should continue monitoring the situation. Meanwhile, COVID-19 vaccination should also continue for control of the pandemic. There are concerns for the ongoing employment of female healthcare workers, and that they be guaranteed safety to continue working in hospitals for gender equity and to increase the taskforce. Additionally, there is a need to ensure that remaining healthcare workers are suitably trained and given resources on how to differentiate between COVID-19 and tuberculosis. Moreover, health promotion programs for the general public, including at-risk population, can help to improve earlier reporting to a health facility and reductions in stigma. . The public health infrastructure needs support in improving the tuberculosis epidemiology and surveillance programs.

In conclusion, the COVID-19 pandemic has shifted the focus of Afghanistan from tuberculosis prevention to mitigating the spread of SARS-CoV-2. Although some of the pandemic response sanitary measures and precautions may be beneficial for the prevention of tuberculosis as well, it is expected that the tuberculosis prevalence will increase. With the humanitarian crisis unfolding, it is important for the national administration to collaborate with international organizations such as UNICEF, WHO and The World Bank to gain their trust in improving the current condition of Afghanistan. The Taliban government should also encourage equal access to healthcare for men and women. Through a multidisciplinary approach, the people of Afghanistan will be able to endure this crisis.

## Ethical approval

N/A.

## Sources of funding for your research

None

## Author contribution

All authors equally contributed.

## Registration of research studies

Name of the registry: N/A.
Unique Identifying number or registration ID: N/A.
Hyperlink to your specific registration (must be publicly accessible and will be checked): N/A

## Consent

N/A.

## Guarantor

N/A.

## Provenance and peer review

Not commissioned, Editor reviewed.

## Declaration of competing interest

None declared.
